# Prioritizing Pediatric Eye Care in Saudi Arabia: A National Delphi Consensus Study

**DOI:** 10.3390/healthcare13192467

**Published:** 2025-09-29

**Authors:** Mansour A. Alghamdi, Ali Almustanyir, Abdulmalik A. Alshuimi, Saif Hassan Alrasheed, Balsam Alabdulkader, Muteb Alanazi, Basal H. Altoaimi, Mohammad Bin Dulaym, Lama Y. Alsamnan, Waleed Alghamdi

**Affiliations:** 1 Optometry Department, College of Applied Medical Sciences, King Saud University, Riyadh 11451, Saudi Arabia; aalmustanyir@ksu.edu.sa (A.A.); 441100272@student.ksu.edu.sa (A.A.A.); alabdulkader@ksu.edu.sa (B.A.); mkalanazi@ksu.edu.sa (M.A.); baltoaimi@ksu.edu.sa (B.H.A.); 2 Department of Optometry, College of Applied Medical Sciences, Qassim University, Buraydah 51452, Saudi Arabia; s.rasheed@qu.edu.sa (S.H.A.); walghamdi@qu.edu.sa (W.A.); 3 King Abdullah bin Abdulaziz University Hospital, Princess Nourah bint Abdulrahman University, Riyadh 11671, Saudi Arabia; msbindlaim@kaauh.edu.sa; 4 King Abdullah Specialized Children’s Hospital, Ministry of National Guard, Riyadh 11426, Saudi Arabia; alsamnanla@mngha.med.sa; 5 School of Optometry and Vision Science, UNSW Sydney, Sydney, NSW 2052, Australia

**Keywords:** Delphi technique, child, quality of life, vision screening, public health

## Abstract

**Background/Objectives:** Childhood eye disorders, including refractive errors, strabismus, and amblyopia, are prevalent yet often underdiagnosed in Saudi Arabia. Limited data on barriers to pediatric eye care hinder efforts to optimize service delivery. This study aimed to identify barriers to accessing pediatric eye care and to develop consensus-based strategies for improvement. **Methods:** A Delphi technique involving three iterative rounds of questionnaires was conducted with a panel of 22 eye care experts across Saudi Arabia. Consensus was defined as ≥80% agreement among participants. In total, 30 statements were developed from thematic analysis of open-ended responses and a supporting literature review. Panelists rated each statement on a five-point Likert scale, and descriptive statistics were applied. Internal consistency across rounds was assessed using Cronbach’s alpha. **Results:** Of the 30 proposed statements, 25 (83.3%) reached consensus, with a mean agreement score of 4.45 ± 0.59. Internal consistency was high (Cronbach’s alpha = 0.92). High-priority recommendations included implementing mandatory vision screening, integrating optometrists into primary healthcare, and establishing specialized pediatric eye care centers. Other recommendations emphasized expanding mobile clinics and increasing public awareness. Areas that did not reach consensus included referral inefficiencies, adequacy of the current workforce, and school accommodations for children with visual impairment. **Conclusions:** This study presents the first national consensus on pediatric eye care in Saudi Arabia and provides actionable recommendations to strengthen services. The findings offer a strategic framework to guide policy, enhance workforce development, and reduce childhood visual impairment through early detection and intervention.

## 1. Introduction

Ophthalmic disorders such as refractive errors, strabismus, amblyopia, and keratoconus are common and often undetected among children in Saudi Arabia [1]. Globally, approximately 20 million children are affected by visual impairment, including 1.4 million who are blind and 17.5 million with severe visual impairment. The majority reside in developing countries in Africa and Asia [2,3]. The Lancet Commission on Global Childhood Eye Health projected that childhood blindness will affect more than 1.02 million children, with a global prevalence of 4.8 per 10,000 children [4].

A recent systematic review and meta-analysis from the Eastern Mediterranean Region estimated the prevalence of visual impairment in children at 11.57%. The leading causes include uncorrected refractive errors (51.89%), amblyopia (11.15%), retinal or congenital disorders (3.90%), corneal opacities (3.00%), and cataracts (1.88%) [5]. Although refractive errors and cataracts are the most common causes, many ophthalmic disorders are preventable or treatable. Early detection and timely management are therefore essential to prevent progression and reduce the risk of irreversible visual impairment or blindness [6,7].

In response to this global need, the World Health Organization (WHO) launched the SPECS 2030 initiative, which aims to increase global access to appropriate spectacles by 40%, specifically targeting preventable visual impairment in children due to uncorrected refractive errors [7].

A study from Saudi Arabia reported that refractive errors, strabismus and amblyopia are the most prevalent ophthalmic disorders among children [1,8]. However, vision screening is typically conducted only upon school entry, which may be insufficient for early detection. Additional barriers hinder access to timely and adequate pediatric eye care services [1,9].

In London, key barriers to pediatric eye care include insufficient parental awareness about how and where to schedule eye examinations, and concerns about overprescription of glasses to children [10]. Wang et al. [11] identified inadequate insurance coverage and scheduling difficulties as primary obstacles among Canadian children. In South India, parental perceptions played a dominant role—71.02% believed their child had no serious eye condition, and 65.12% assumed that adequate daily functioning indicated normal vision [12]. A previous Delphi study on major incident planning found that pediatric services are crucial for both preparedness and response when children are involved [13]. Recent studies [14,15,16,17] found that myopia, the most common refractive error, is linked to increasing age, urban living, and childhood visual impairment. They recommended strengthening school eye health programs to enhance refractive services and support progress toward targets. Furthermore, a recent study in Ethiopia identified gaps in child eye health services and emphasized the need for stronger health policies, sustainable financing, and collaboration with key stakeholders to improve service delivery [18].

Despite the global recognition of these issues, limited data are available on the specific barriers to pediatric eye care in Saudi Arabia. To address this gap, the present study aimed to identify key obstacles to accessing eye care for children and to propose consensus-based strategies aimed to improve service delivery and accessibility.

## 2. Materials and Methods

### 2.1. Study Design

This study used the classical Delphi technique, a structured exploratory method that involves administering multiple rounds of questionnaires to a panel of experts. The process continued until a predefined level of consensus was reached [19,20]. In this study, the technique was applied to achieve consensus on a series of statements initially developed through a comprehensive literature review. Additional statements were incorporated based on feedback from the expert panel. Consensus level was defined as ≥80% agreement among participants.

### 2.2. Participant Selection

Participants were selected from both the private and governmental sectors and were actively involved in eye care in Saudi Arabia. The panel was intentionally composed to ensure balanced representation from these two stakeholder groups. A total of 22 experts were identified and invited to participate. Invitations and informed consent forms were distributed via email, followed by telephone calls to confirm receipt and encourage participation. These efforts were conducted two weeks before the initiation of the Delphi process.

### 2.3. Inclusion Criteria

Participants were required to be eye care professionals with at least four years of experience in the field. Individuals with fewer than four years of experience or with professional backgrounds unrelated to eye care were excluded.

### 2.4. Ethical Approval

Ethical approval was obtained from the institutional review board prior to participant recruitment (IRB approval number: E-23-7479). The study was conducted in accordance with the Declaration of Helsinki. Participant confidentiality was strictly maintained, and all participants provided a written informed consent form before participation in the Delphi rounds.

### 2.5. Panel Size

While no universally accepted standard exists for the ideal size of a Delphi panel, prior research suggests that 20–50 experts are typically appropriate [20]. Accordingly, this study included 22 qualified eye care professionals who consented to participate.

### 2.6. Delphi Process

Each panelist initially received an information letter and informed consent form via email. The letter explained the study’s aims, purpose of participation, and the right to withdraw. Panelists were asked to return a signed consent form to confirm participation. The invitation also included a request for demographic data, such as age, sex, employment organization, years of experience, qualifications, and professional role.

### 2.7. Delphi Statement Development

An open-ended questionnaire was used to gather panelists’ perspectives on barriers to childhood eye care and to collect recommendations for improving eye care services in Saudi Arabia. Participants were asked to provide detailed responses, including ideas, approaches, and suggested solutions. The questionnaire addressed six broad themes:

1. Barriers to accessing childhood eye care.
2. Services and instruments for children with visual impairment.
3. Actions needed to develop a national childhood eye care plan.
4. Challenges faced by families of children with visual impairment.
5. Strategies to improve childhood eye care services.
6. Interventions to overcome access barriers.

Responses were reviewed and organized into six themes, and initial Delphi statements were generated through thematic analysis of participant input. These were then refined and supplemented by a comprehensive literature review on pediatric eye care priorities, challenges, and service gaps in Saudi Arabia. Previous studies have highlighted workforce shortages and uneven distribution of services [9,20], delays in access and pressure on public healthcare systems [21], and global recommendations for integrated, equitable strategies [22,23]. Aligning these findings with participant responses ensured that the questionnaire was context-specific, evidence-based, and concise. To maintain balance and feasibility, five statements were selected per theme. This approach ensured that the questionnaire was comprehensive yet manageable, and is consistent with previous Delphi studies in pediatric eye care [20,24]

A pilot study was conducted by sending the drafted statements to a separate group of eye care professionals not involved in the original Delphi panel. These individuals reviewed the statements and provided feedback on clarity and potential errors.

#### 2.7.1. Round One

The first-round Delphi questionnaire, developed in Microsoft Excel, was distributed via email to 22 consenting experts. It included the consent form and an instruction letter. Panelists were asked to rate each statement using a five-point Likert scale (“strongly agree” to “strongly disagree”) and provide written comments. Written comments provided by panelists during rounds were carefully reviewed. These qualitative insights helped refine the wording of existing statements, identify areas of ambiguity, and highlight emerging themes not previously captured. Statements were revised or added accordingly to ensure they reflected expert perspectives and improved clarity and relevance in the following round. Completed questionnaires were returned via email within one week. All 22 panelists responded. Statements reaching ≥80% agreement were considered to have achieved consensus. This round took two weeks to collect participant responses and complete the analysis.

#### 2.7.2. Round Two

The second round included a summary of first-round results. Statements that did not reach consensus in the first round and new statements were returned for further review. Each panelist received a personalized questionnaire showing their own responses alongside group-level data. An instruction letter was included to guide panelists’ completion. Participants were asked to reconsider their responses and indicate any changes. All 22 panelists from the first round completed the second round. This round took two weeks to collect participant responses and complete the analysis.

#### 2.7.3. Round Three

**Figure 1:**
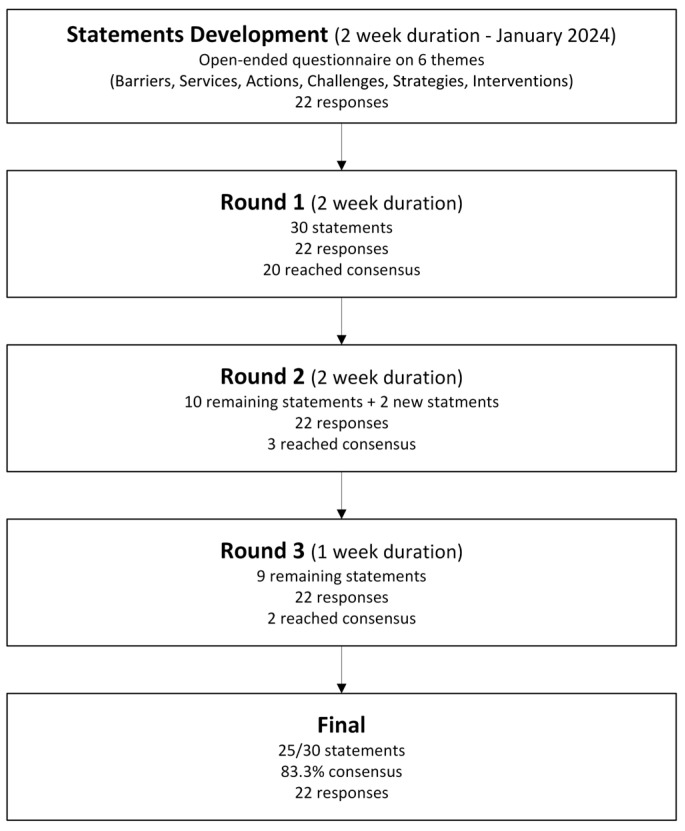
Flowchart of the Delphi process. A 2-week statement development phase generated items across six themes, followed by three rounds with 22 experts. Of 30 initial statements, 25 (83.3%) achieved consensus by the final round.

In the third and final round, a summary of the second-round results and consensus levels was shared. Statements that had reached ≥80% agreement were excluded. Remaining items and newly generated statements were resubmitted to panelists for final review. Participants were asked to confirm their rankings and indicate whether no further changes were needed. This was interpreted as evidence of response stability. Final results from all rounds were compiled into a consensus statement. This round took three weeks to collect participant responses and complete the analysis. The Delphi process, from statement development through three rounds to the final consensus, is shown in Figure 1.

### 2.8. Analysis

Data from all Delphi rounds were entered into Microsoft Excel. Descriptive analysis was performed, and consensus was defined as ≥80% agreement among panelists.

## 3. Results

Table 1 summarizes the demographic characteristics of the expert panel. A total of 22 experts (14 men and 8 women) participated, with a mean age of 41.5 years (range: 29–58 years) and an average of 13.5 years of professional experience (range: 5–30 years). The panel comprised 13 optometrists and 8 ophthalmologists, holding a range of academic qualifications, including bachelor’s, master’s, and doctoral degrees, as well as professional fellowships. Participants represented both the governmental and private healthcare sectors across Saudi Arabia.

Experts were geographically distributed according to population density and willingness to participate, with the highest concentration from Riyadh (n = 13), followed by the Western Region (Jeddah, n = 3), Eastern Region (Dammam, n = 2), Southern Region (n = 2), and Northern Region (n = 2).

Based on the six previously identified themes, 30 Delphi statements (five per theme) were developed and refined over three rounds. Participants rated their agreement with each statement using a 5-point Likert scale. All 22 panelists completed all three Delphi rounds (100% response rate). Agreement percentages were calculated using the following formula:

(1) $$Agreement\;Percentage=(\frac{Mean\;Score}{5})\times 100$$

Consensus was defined as ≥80% agreement. Of the 30 statements, 25 (83.3%) reached consensus, with a mean agreement score of 4.45 ± 0.59. Internal consistency was high, with a Cronbach’s alpha of 0.92. The 25 statements were endorsed by the panel to inform the development of a childhood eye care plan in Saudi Arabia.

Table 2 presents the final consensus items, including consensus percentages together with the mean ± standard deviation (SD) and the median with interquartile range (IQR) to provide a detailed description of expert ratings.

**Figure 2:**
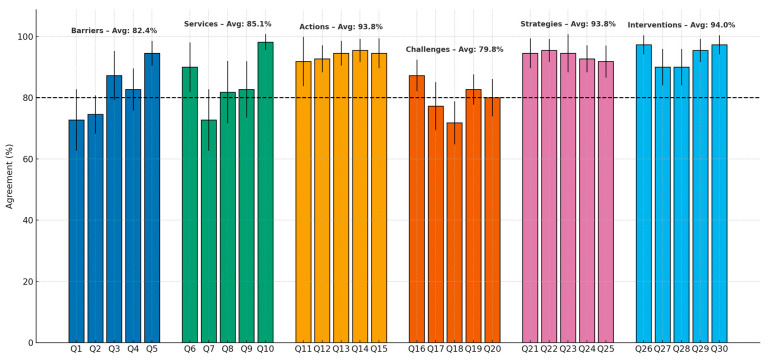
Agreement levels for Delphi statements (Q1–Q30) grouped into six themes: Barriers (Q1–Q5), Services (Q6–Q10), Actions (Q11–Q15), Challenges (Q16–Q20), Strategies (Q21–Q25), and Interventions (Q26–Q30). Error bars show 95% confidence intervals, and the dashed line marks the 80% consensus threshold. Theme averages are displayed above each group.

Figure 2 illustrates agreement levels across all themes, highlighting the number of statements that reached consensus (≥80%) and those that did not.

## 4. Discussion

This Delphi study focused on pediatric eye care in Saudi Arabia and reached consensus on key actions to improve services for children. High-priority recommendations included implementing mandatory vision screening programs, integrating optometrists into primary healthcare, and expanding access to specialized pediatric eye care through dedicated centers and clinics. While consensus (≥80% agreement) was reached for most statements, areas such as referral systems, specialist shortages, and school accommodations require further attention. Building on this finding, the study offers a roadmap for policymakers and healthcare providers to strengthen pediatric eye care in Saudi Arabia. The consistency of responses was further supported by distribution measures, which confirmed that expert ratings were highly clustered, indicating strong agreement.

### 4.1. High Consensus Areas

1. Integration of optometrists: Strong agreement was observed regarding the integration of optometrists into primary healthcare and the expansion of their roles.
2. Mandatory school vision screening: There was unanimous support for implementing mandatory vision screening programs, particularly those aligned with vaccination schedules and school entry.
3. Workforce development: High consensus emphasized the need to train and recruit additional pediatric eye care specialists.
4. Public awareness: Participants supported launching nationwide campaigns to increase awareness of pediatric eye health.
5. Infrastructure expansion: There was agreement on the need to establish specialized pediatric eye care clinics and introduce mobile eye clinics to improve accessibility.

### 4.2. Areas Requiring Further Attention (<80% Agreement)

1. Shortage of pediatric eye care specialists (72.86%): Some participants may have perceived the current workforce as sufficient or had differing views regarding specialist distribution.
2. Challenges with the government referral system (74.29%): Opinions varied concerning the complexity and usability of the referral system for parents.
3. Need for specialized diagnostic tools (72.86%): Differences emerged regarding the necessity and prioritization of advanced tools such as photoscreeners and autorefractors.
4. Access difficulties for families (77.14%): Participants reported varying experiences and perceptions with families’ ability to access specialized eye care.
5. School accommodations for children with visual impairment (71.43%): Disparities in school resources across regions may have influenced participant responses.

The Delphi process identified key barriers to childhood eye care access, with strong expert consensus on three major issues: the shortage of ophthalmologists and optometrists in primary care, the lack of structured school screening programs, and the burden on public healthcare services. The shortage of eye providers limits early detection and referral, particularly in underserved areas, as also noted by Alrasheed et al. [20]. Similarly, the absence of organized school screening programs undermines early intervention, despite evidence supporting their effectiveness in reducing avoidable pediatric visual impairment [25]. Moreover, the pressure on public services contributes to delayed diagnosis and treatment, a persistent issue in regional health system evaluations [21].

In Saudi Arabia, citizens receive free healthcare, with the Ministry of Health delivering most services through its nationwide hospitals and health centers. Since 2002, mandatory health insurance has also been phased in under the Health Insurance Council. Although universal coverage is a strength, rapid population growth and longer life expectancy have strained the system, resulting in long waits for many services, including eye care. For children with vision problems, access often begins with a referral from primary care to an eye clinic, a step that can delay timely diagnosis and treatment. Families frequently seek private clinics to bypass these delays, paying out of pocket. These structural characteristics, which differ from Western healthcare systems such as the United States, help contextualize the panel’s concerns about waiting times and referral processes and likely influence the implementation of pediatric eye care strategies in Saudi Arabia.

A recent study in Saudi Arabia reported that services such as pediatric optometry clinics, dedicated ophthalmology outpatient units, and advanced diagnostic tools were more widely available in the Eastern region than elsewhere [9]. In contrast, a previous study conducted in Sudan, part of the Eastern Mediterranean Region, used Delphi methods to build consensus on developing child eye care services. Similarly, a recent study in England identified communication issues and the lack of suitable equipment for examining children as key barriers to accessing primary eye care [24]. The study identified key barriers to pediatric eye care, including financial constraints, limited clinical access, and lack of public awareness. It recommended stronger collaboration between the Ministries of Health and Education, along with non-governmental organizations (NGOs), to effectively address these challenges [20]. A systematic review in Sub-Saharan Africa found that poverty, low education, weak healthcare systems, and a shortage of professionals are key barriers to accessing healthcare services [26]. In contrast, access to eye care in Pakistan is hindered by several key barriers, including illiteracy, long travel distances, high costs, female gender, old age, and mobility limitations [27]. These factors collectively reduce the likelihood of individuals seeking or receiving timely eye care services. Building on this finding, addressing these barriers requires strategic investment in workforce development, school-based health initiatives, and healthcare infrastructure to ensure equitable and timely pediatric eye care.

The Delphi results also showed strong consensus on essential instruments and services for managing childhood visual impairment. Priorities included cycloplegic eye drops and diagnostic agents, basic optometry equipment, vision therapy services, and low-vision devices. These findings align with previous studies emphasizing the importance of equipping pediatric eye care services with appropriate tools for accurate diagnosis and intervention [9,20,22]. Similarly, the American Academy of Ophthalmology reports that instrument-based screening methods like photoscreening and autorefraction are effective for detecting amblyopia risk factors in children aged 1–5, especially during critical stages of visual development. These tools are also useful for older children and those with developmental disabilities who may struggle with standard vision tests [28]. Zhu et al. [29] highlighted the role of cycloplegic refraction in detecting refractive errors in children. The WHO similarly recommends integrating vision rehabilitation and assistive technologies into pediatric health services to reduce the burden of visual impairment and support inclusive education [21].

The success of a national pediatric eye care plan depends on strategies endorsed by the Delphi panel, including the integration of optometrists into primary care, mandatory school vision screening with robust referral systems, and regular community-based vision screenings. Similarly, the American Academy of Pediatrics recommends vision screening from birth as part of routine health checks. Early screening before age 7 can reduce causes of blindness like amblyopia by up to 50%, and regular assessments through adolescence help detect vision problems early [30]. The WHO underscores the importance of integrating optometrists in primary care for early detection and management of refractive errors in children [23]. School vision screening, when supported by effective referral and follow-up systems, enables timely diagnosis and management of visual impairment, and reduces preventable childhood blindness [20,23]. Community-based screening is also critical for reaching underserved populations, including children not enrolled in school, and is a central pillar of WHO’s SPECS 2030 initiative to promote equitable access to pediatric eye care services [7]. Together, these strategies offer a comprehensive and sustainable framework for improving pediatric eye health outcomes. A recent study in Malawi suggested that school-based eye care can be supported by teachers, community informants, and health workers through vision screening, raising awareness of the impact of visual impairment, and reducing stigma around wearing spectacles [31].

The Delphi results demonstrated consensus that children with visual impairment face several persistent challenges, including the high cost of visual aids, social stigma, isolation, and a lack of family awareness regarding appropriate eye care services. These findings are consistent with recent global reports on children with developmental disabilities, which have emphasized that such children often encounter systemic neglect, stigmatization, and barriers to healthcare access [32,33]. These reports underscore the need for inclusive health systems that address both financial and social determinants of access. Similarly, previous studies [20,25] have highlighted the importance of equitable access to eye care for vulnerable populations and have advocated for community-based strategies to reduce preventable childhood blindness. Addressing these multifactorial challenges will require coordinated efforts across health, education, and social sectors to ensure timely, affordable, and stigma-free eye care for children with visual impairment.

To enhance pediatric eye care services, experts strongly recommended implementing nationwide awareness campaigns, deploying mobile eye clinics, and establishing specialized pediatric eye care clinics. Awareness campaigns have been shown to improve public understanding of pediatric eye conditions and the importance of early detection, contributing to increased screening and treatment uptake [34,35,36]. Mobile eye clinics can effectively reach underserved areas by providing on-site screenings, refractions, and referrals, thereby minimizing geographic and financial barriers. Specialized pediatric eye clinics offer targeted services delivered by trained professionals, improving diagnostic accuracy and clinical outcomes. These approaches align with WHO recommendations for integrated and equitable eye care systems and have shown success in reducing preventable childhood blindness in various global contexts [4,23]. The WHO and International Agency for the Prevention of Blindness (IAPB) recommend increasing public education to raise awareness among parents and teachers, and encouraging 2–3 h of outdoor time daily for schoolchildren as a practical intervention to help prevent and slow the progression of epidemic childhood myopia [37].

The Delphi panel further recommended addressing access barriers in Saudi Arabia by increasing the number of optometry clinics within primary healthcare centers, expanding training programs for pediatric eye care specialists, and establishing specialized services in rural regions to support equitable access. These initiatives can be operationalized through infrastructure upgrades, specialist training, deployment of mobile clinics, and adoption of telemedicine to reach underserved communities. A recent study assessing childhood eye care services in Saudi Arabia found that the eastern region had the strongest capacity, with 95% of hospitals having trained pediatric teams and 88% employing pediatric nurses. It also had superior infrastructure, including dedicated clinics and advanced equipment. In contrast, the southern region had the lowest percentage of trained eye care professionals (44%). The study revealed regional discrepancies in childhood eye care services and facility types, highlighting areas that may require attention from Ministry of Health decision-makers [9]. Furthermore, a systematic review in Africa identified affordability, accessibility, and availability as major barriers to pediatric eye care. The study recommended addressing these challenges requires active involvement from all relevant stakeholders [22].

Although the Delphi method effectively identified priorities, several limitations should be acknowledged. First, while the expert panel was diverse, it included only 22 participants across all three rounds, which may fully reflect the perspectives of providers underserved or rural areas. Furthermore, study included only representatives from the eye care profession and did not involve other key stakeholders such as policymakers, parents/families, health managers, or other healthcare service providers. Second, the prioritization process, though structured, relied on expert opinion and may introduce subjectivity. Third, the geographical focus of this study limits the generalizability of its findings to other healthcare systems. Lastly, while the recommendations provide a robust framework, real-world implementation may be constrained by resource limitations, policy barriers, and challenges in stakeholder coordination. Future research should include broader and more geographically representative panels and assess the feasibility and impact of implementing these strategies in practice.

## 5. Conclusions

This Delphi study established expert consensus on the development of pediatric eye care services in Saudi Arabia. Key barriers identified included limited parental awareness, insufficient access to specialized care, and gaps in school-based vision screening programs. The findings have important policy implications, highlighting the need for mandatory school vision screening, integration of optometrists into primary healthcare, and the establishment of specialized pediatric eye care centers. Strengthening collaboration between health and education sectors, alongside support from NGOs, is essential for effective implementation. While strong consensus (≥80%) was reached for many recommendations, aspects such as referral system improvements and the shortage of specialists warrant further investigation. Addressing these challenges can support the development of inclusive, effective pediatric eye care services in Saudi Arabia, informing global efforts to reduce childhood visual impairment through early detection and intervention. Future research should focus on evaluating the impact of these interventions, exploring innovative service delivery models, and addressing workforce shortages to ensure sustainable and inclusive pediatric eye care.

## Figures and Tables

**Table 1:** Sex, Profession, and Qualifications of Panel Participants (n = 22).

Profession	Qualification	Male	Female	Total
Optometrists	OD/BSc in Optometry	5	3	8
	MSc in Optometry	2	1	3
	PhD in Optometry	2	1	3
Ophthalmologists	Fellowship in Ophthalmology	4	2	6
	PhD in Ophthalmology	1	1	2
Total		14	8	22

**Table 2:** Final consensus on prioritizing pediatric eye care in Saudi Arabia.

Themes and Related Delphi Statements	Consensus Level (%)	Mean ± SD	Median (IQR)
Barriers to accessing childhood eye care			
Lack of optometrists in primary healthcare centers	94.29%	4.73 ± 0.46	5 (4–5)
Lack of qualified school-based screening programs	87.14%	4.36 ± 0.90	4 (4–5)
Overburdened public healthcare systems	82.86%	4.14 ± 0.77	4 (4–5)
2. Services and instruments for children with visual impairment			
Availability of cycloplegic eye drops and diagnostic agents	98.57%	4.91 ± 0.29	5 (5–5)
Availability of basic optometry instruments	90.00%	4.50 ± 0.91	5 (4–5)
Availability of vision therapy tools	82.86%	4.14 ± 1.04	4 (4–5)
Availability of low vision aids	81.43%	4.09 ± 1.15	4 (4–5)
3. Actions needed to develop a national childhood eye care plan			
Employing optometrists in primary care settings	95.71%	4.77 ± 0.43	5 (5–5)
Establishing an efficient referral system	94.29%	4.73 ± 0.46	5 (4–5)
Implementing a centralized monitoring system	94.29%	4.73 ± 0.55	5 (5–5)
Mandating school vision screening	92.86%	4.64 ± 0.49	5 (4–5)
Conducting regular vision screenings for the community	91.43%	4.59 ± 0.91	5 (4–5)
4. Challenges faced by families of children with visual impairment			
High cost of visual aids	87.14%	4.36 ± 0.58	4 (4–5)
Stigma and social isolation	82.86%	4.14 ± 0.56	4 (4–4)
Uncertainty about where to seek care	80.00%	4.00 ± 0.69	4 (4–4)
5. Strategies to improve childhood eye care services			
Vision screening at key developmental milestones	95.71%	4.77 ± 0.43	5 (5–5)
Nationwide public awareness campaigns	94.29%	4.73 ± 0.55	5 (5–5)
Recognition of optometrists as primary care providers	94.29%	4.73 ± 0.70	5 (5–5)
Deployment of mobile eye clinics	92.86%	4.64 ± 0.49	5 (4–5)
Establishment of specialized pediatric eye care clinics	91.43%	4.59 ± 0.59	5 (4–5)
6. Interventions to overcome access barriers			
Expanding optometry services in primary health centers	97.14%	4.86 ± 0.35	5 (5–5)
Training more pediatric eye care specialists	97.14%	4.86 ± 0.35	5 (5–5)
Including vision screening in the child health passport	95.71%	4.77 ± 0.43	5 (5–5)
Increasing the number of pediatric eye care clinics	90.00%	4.50 ± 0.67	5 (4–5)
Providing specialized services in rural areas	90.00%	4.50 ± 0.67	5 (4–5)

Note: Median (IQR) represents the median score and interquartile range (25th–75th percentile).

## Data Availability

The datasets used and analyzed during the current study are available from the corresponding author on reasonable request.

## Abbreviations

The following abbreviations are used in this manuscript:

WHO	World Health Organization
IRB	Institutional review board
OD	Optometry doctor
